# Sense of Purpose in Life Promotes Resilience to Memory Deficits Related to Depressive Symptomatology

**DOI:** 10.1093/geroni/igab046.982

**Published:** 2021-12-17

**Authors:** Nathan Lewis, Patrick Hill

**Affiliations:** 1 University of Victoria, Victoria, British Columbia, Canada; 2 Washington University in St. Louis, St. Louis, Missouri, United States

## Abstract

Individuals higher in depressive symptoms commonly present with neuropsychological deficits including poorer memory performance. Sense of purpose in life, a component of psychological well-being, has been shown to promote resilience to cognitive impairment in older adulthood, but it is unclear whether it may also protect against cognitive deficits associated with higher depressive symptoms. This study examined whether purpose in life moderated the effect of depressive symptoms on cognitive functioning in a large longitudinal study of 4599 American older adults (Mage = 74.33 years, range = 65–104 years, 56.84% female) across a 12-year follow-up period. Depressive symptomatology was assessed at each wave using the 8-item Center for Epidemiologic Studies Depression Scale. Multilevel models assessed the influence of depressive symptoms and the interaction with sense of purpose in life on changes in word recall and mental status. Higher depressive symptoms were associated with poorer recall at baseline, but not rate of change over time. A negative interaction was observed between sense of purpose in life and depressive symptoms such that individuals higher in purpose experienced a less negative impact of depressive symptoms on word recall. No significant interaction of sense of purpose and depressive symptoms was observed on mental status. Having a sense of purpose in life may help protect older adults from memory deficits associated with higher depressive symptoms. The present findings underscore the potential for sense of purpose to promote cognitive reserve in older adulthood, allowing individuals to maintain cognitive performance in the face of accruing neuropsychological challenges.

